# Nurse Visits, Site of Care, and Hospitalization Among Hospice Patients With Dementia

**DOI:** 10.1093/geroni/igaa057.1595

**Published:** 2020-12-16

**Authors:** Elizabeth Luth, David Russell, Holly Prigerson, Kathryn Bowles, Miriam Ryvicker

**Affiliations:** 1 Weill Cornell Medical College, Larchmont, New York, United States; 2 Appalachian State University, Boone, North Carolina, United States; 3 Weill Cornell Medicine, New York, New York, United States; 4 University of Pennsylvania School of Nursing, Philadelphia, Pennsylvania, United States; 5 Visiting Nurse Service of New York, New York, United States

## Abstract

Persons with dementia comprise up to 50% of hospice patients and face an increased risk of burdensome, disruptive, and costly discharge from hospice due to hospitalization. The relationship between timing, dose and site of hospice care provided, all modifiable factors, and risk of hospitalization is poorly understood. We use a retrospective cohort analysis of 2,692 electronic health records of hospice patients with dementia who received care from a large hospice agency in New York City between 2013-2017 to determine the relationship between hospice service delivery (e.g., number and timing of nurse visits, home vs. facility-based) and risk of hospitalization (vs death). We control for demographic and clinical characteristics of patients. 9.36% of patients with dementia were hospitalized. Hospice service delivery factors were significantly associated with risk of hospitalization. Each additional nurse visit was associated with a 5% decrease in risk of hospitalization (AOR: 0.95, 95% CI: 0.92-0.98). Each additional day between last nurse visit and discharge was associated with a 7% increase in risk of hospitalization (AOR: 1.07, 95% CI: 1.04-1.11). Home hospice was associated with 97% higher odds of hospitalization (AOR: 1.97, 95% CI: 1.19-2.09). Hospice patients with dementia who receive services at home, receive fewer nursing visits, and have increased time between nursing visits are at increased risk for hospitalization. Research is needed to determine if increasing the number and timing of nursing visits can reduce risk of hospitalization in this population.

